# A high‐fat diet reverses metabolic disorders and premature aging by modulating insulin and IGF1 signaling in SIRT6 knockout mice

**DOI:** 10.1111/acel.13104

**Published:** 2020-01-22

**Authors:** Zhongchi Li, Kang Xu, Yannan Guo, Lu Ping, Yuqi Gao, Ying Qiu, Jianquan Ni, Qingfei Liu, Zhao Wang

**Affiliations:** ^1^ Protein Science Key Laboratory of the Ministry of Education School of Pharmaceutical Sciences Tsinghua University Beijing China; ^2^ 8‐year MD Program Peking Union Medical College Beijing China; ^3^ School of Medicine Tsinghua University Beijing China

**Keywords:** fatty acid, glycolysis, high‐fat diet, insulin/IGF1 signaling, organ atrophy, SIRT6

## Abstract

Mammalian sirtuin 6 (SIRT6) is involved in the regulation of many essential processes, especially metabolic homeostasis. SIRT6 knockout mice undergo premature aging and die at age ~4 weeks. Severe glycometabolic disorders have been found in SIRT6 knockout mice, and whether a dietary intervention can rescue SIRT6 knockout mice remains unknown. In our study, we found that at the same calorie intake, a high‐fat diet dramatically increased the lifespan of SIRT6 knockout mice to 26 weeks (males) and 37 weeks (females), reversed multi‐organ atrophy, and reduced body weight, hypoglycemia, and premature aging. Furthermore, the high‐fat diet partially but significantly normalized the global gene expression profile in SIRT6 knockout mice. Regarding the mechanism, excessive glucose uptake and glycolysis induced by the SIRT6 deficiency were attenuated in skeletal muscle through inhibition of insulin and IGF1 signaling by the high‐fat diet. Similarly, fatty acids but not ketone bodies inhibited glucose uptake, glycolysis, and senescence in SIRT6 knockout fibroblasts, whereas PI3K inhibition antagonized the effects of a high‐fatty‐acid medium in vitro. Overall, the high‐fat diet dramatically reverses numerous consequences of SIRT6 deficiency through modulation of insulin and IGF1 signaling, providing a new basis for elucidation of SIRT6 and fatty‐acid functions and supporting novel therapeutic approaches against metabolic disorders and aging‐related diseases.

## Graphical abstract

SIRT6 deficiency induces hyper activation of insulin and IGF1 signaling, accompanied with excessive anaerobic glycolysis and premature aging phenotypes. High‐fat diet effectively extends the lifespan and rescues mutant phenotypes in SIRT6 knockout mice. Fatty acid but not ketone bodies inhibit glucose uptake and anaerobic glycolysis induced by SIRT6 deficiency.

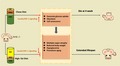

## 1 INTRODUCTION

Sirtuins, as a highly conserved family of NAD^+^‐dependent deacetylases, are a class of molecules exerting broad influence on numerous biological pathways (Finkel, Deng, & Mostoslavsky, 2009). SIRT6 was reported to play a key part in the regulation of metabolism affecting aging and tumorigenesis (Mostoslavsky et al., 2006; Sebastian et al., 2012). SIRT6 knockout (KO) mice manifest acute degenerative and aging‐like abnormalities and succumb to death by 4 weeks of age. Before death, these mice have a smaller‐than‐normal body size in combination with lymphopenia, osteoporosis, and muscle atrophy, which resembles aging‐induced sarcopenia and frailty (Mostoslavsky et al., 2006; Samant, Kanwal, Pillai, Bao, & Gupta, 2017). Furthermore, SIRT6 was found to regulate glucose homeostasis because SIRT6 deficiency induces excessive glucose uptake and glycolysis and reduces mitochondrial respiration (Zhong et al., 2010). Alterations in glycometabolism have been proposed to underlie the diverse aberrations associated with SIRT6 deficiency (Zhong et al., 2010), and many aging‐related diseases derive from chronic metabolic problems in our daily life. Whether direct alteration of glycometabolism via a diet‐based intervention could reverse any SIRT6 KO‐induced problems remains to be tested.

Insulin and IGF signaling pathways are deeply involved in anabolism and catabolism, which largely determine the metabolic homeostasis of the whole body (Haeusler, McGraw, & Accili, 2018; Taniguchi, Emanuelli, & Kahn, 2006). Two‐way abnormal changes in insulin sensitivity are usually found in some metabolically morbid states, such as obesity, diabetes, cancer, and aging (Fujita, Hayashi, Matsushita, Uemura, & Nonomura, 2019; Santoleri & Titchenell, 2018; Sattler, 2013; Siddle, 2012). More and more studies shed new light on the role of metabolites as regulators of insulin sensitivity and metabolism (Yang, Vijayakumar, & Kahn, 2018). Fatty acids were found to perform a regulatory function in many biological processes not merely serve as an energy source (Papackova & Cahova, 2015). Fatty acids may inhibit insulin signaling activity in multiple ways, and these effects are dependent on the fatty‐acid type (Holland et al., 2011; Yang et al., 2018).

Here, we tested the influence of a diet‐based intervention on SIRT6 KO‐induced abnormalities and tried identifying the underlying mechanism. After treatment of the mice with a high‐fat diet, we observed a striking reversal of numerous seemingly diet‐unrelated aberrations; this reversal included increased body size, attenuation of multi‐organ atrophy, improved blood glucose and lipid levels, and decreased blood lactate level, which is increased in old animals (Ross et al., 2010). As for the mechanism, we show that overactivation of insulin and IGF1–PI3K signaling caused by the SIRT6 deficiency is dramatically attenuated by the high‐fat diet. In contrast, a supplement of 10% glucose (in water) did not reverse these SIRT6 KO‐induced aberrations. These results emphasize that insulin and IGF signaling cascades have more functions than those already known. Furthermore, fatty acids but not ketone bodies inhibited excessive glycolysis and senescence caused by the SIRT6 deficiency, and these problems were found to be PI3K dependent. These results offer insights into the pleiotropy of SIRT6 KO‐induced anomalies and have implications for the application of diet‐based interventions against aging and aging‐related diseases.

## 2 RESULTS

### 2.1 The high‐fat diet but not a glucose supplement significantly extends the lifespan of SIRT6 KO mice

**Figure 1:**
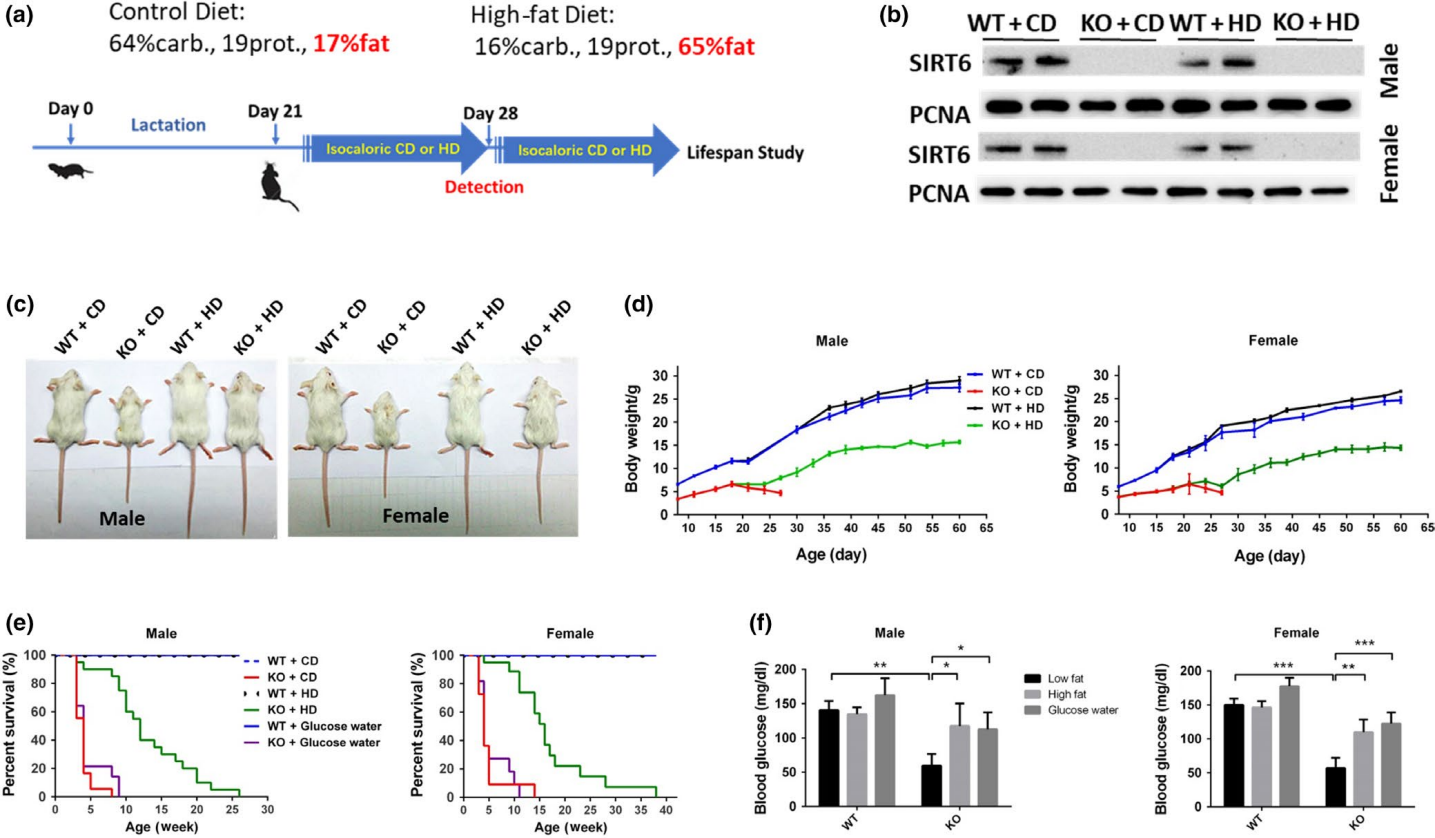
The high‐fat diet significantly extends the lifespan of SIRT6 KO mice. (a) According to the different genotypes and diets, the mice were subdivided into four groups, including SIRT6 knockout (KO) mice and wild‐type (WT) littermates treated with the high‐fat diet (groups WT + HD and KO + HD, respectively) or normal control diet (groups WT + CD and KO + CD, respectively). The time points in this process are indicated. (b) The expression of SIRT6 in the liver from both male and female mice was tested by Western blotting, and PCNA served as a reference. (c) The appearances of mice in different groups at 4 weeks of age. (d) The body weight was measured every 3–5 days starting at 1 week after the mice were born (*n* = 10–15). (e) The numbers of deaths in different groups of mice were recorded to build a survival curve (*n* = 15–30). (f) The blood glucose levels in both male and female mice were tested after 3 hr fasting (*n* = 6). Data are presented as mean ± *SD*. *p < .05, **p < .01, ***p < .001. See also Figure S1

The SIRT6 KO mice and wild‐type (WT) littermates were treated with a high‐fat diet (groups WT + HD and KO + HD, respectively) or a normal control diet (groups WT + CD and KO + CD, respectively) since 3 weeks of age (Figure 1a). Western blot analysis of total liver protein showed that SIRT6 was completely absent in the KO groups, and the high‐fat diet treatment did not affect the expression of SIRT6 (Figure 1b). At 4 weeks, based on appearance, the body size of male and female mice was obviously increased as a result of the consumption of high‐fat diet (Figure 1c). To evaluate the effect of the prolonged high‐fat diet on SIRT6 KO mice, the mice were treated with the high‐fat diet from 3 weeks of age until death. The body weight of KO mice started to decrease since ~3 weeks of age, and the animals usually died when the body weight reached ~6 grams but the body weight of KO + HD mice recovered from the bottom and kept increasing after survival (Figure 1d). Prolonged observation suggested that KO + HD mice had a strikingly longer lifespan as compared to KO + CD mice (males showed a 5.5‐fold increase in the maximum lifespan, and female mice manifested an 8‐fold increase in the maximum lifespan; Figure 1e). Hypoglycemia has been reported to be the direct cause of the early death of SIRT6 KO mice (Mostoslavsky et al., 2006), and the high‐fat diet effectively increased the blood glucose level and triglyceride level in both male and female SIRT6 KO mice (Figure 1f). By contrast, supplementation with 10% glucose in water did not prevent early death, even though the blood sugar level increased (Figure 1e,f). There were no remarkable differences in total cholesterol (CHO) between WT and KO mice, but the high‐density lipoprotein cholesterol/ CHO ratio decreased while the low‐density lipoprotein cholesterol/CHO ratio increased as a result of the SIRT6 deficiency; these changes were significantly attenuated by the high‐fat diet only in male mice (Figure S1). These results suggested that the high‐fat diet but not the glucose supplement extended the lifespan and reversed the low body weight of SIRT6 KO mice.

### 2.2 The high‐fat diet reverses the SIRT6 KO‐induced atrophy and premature aging features

The SIRT6 KO induced atrophy of multiple organs and tissues concomitantly with the decreased body size, whereas the high‐fat diet did attenuate the atrophy of the heart, liver, spleen, thymus, kidneys, white adipose tissue, and intestines both in males and females. Besides, total fat mass and bone mineral density were increased by the high‐fat diet only in KO mice (Figure S2, Table S1).

**Figure 2:**
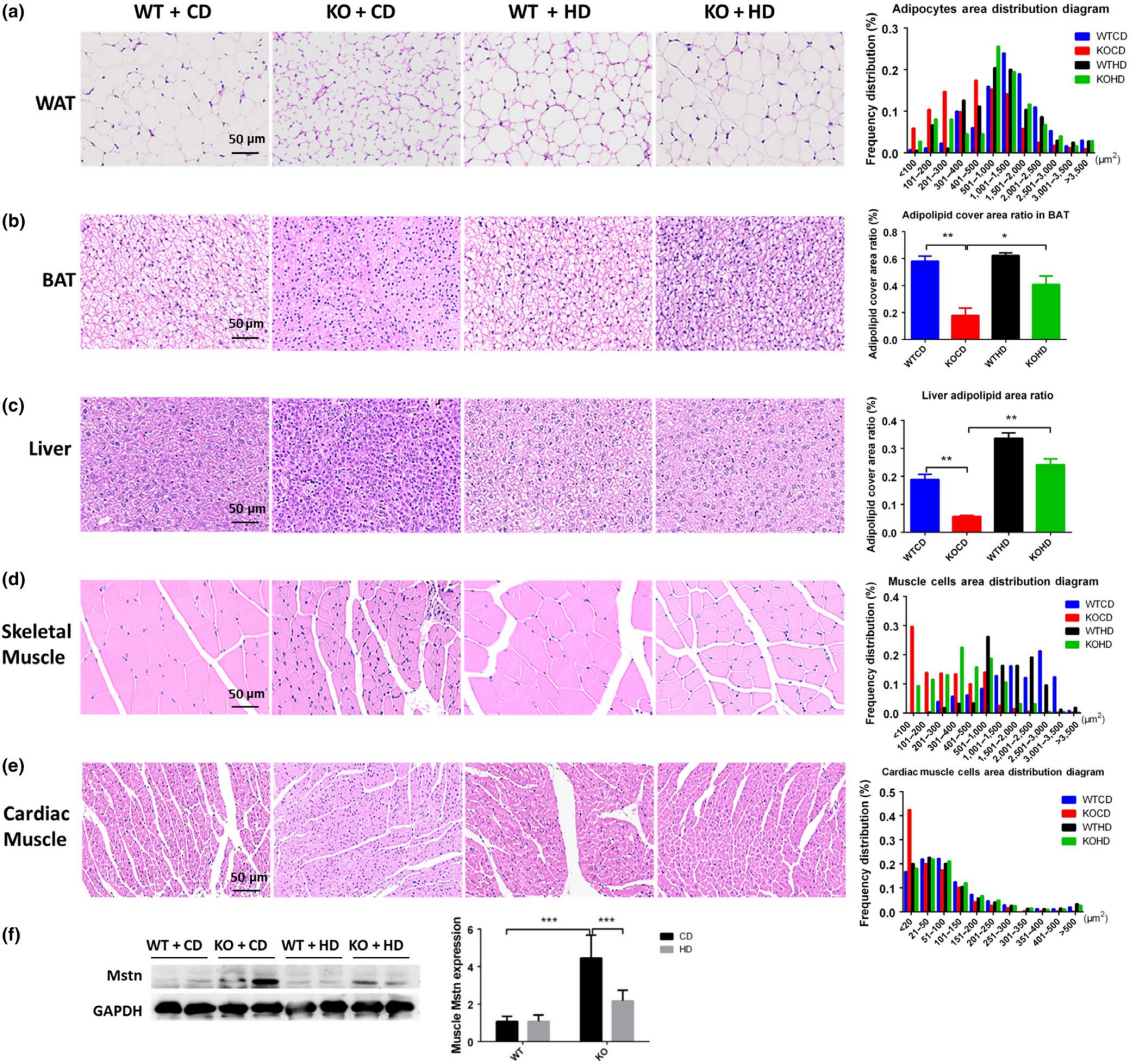
The high‐fat diet reverses the SIRT6 KO‐induced multi‐organ and multi‐tissue atrophy features. Epididymal white adipose tissue (a), BAT (b), the liver (c), skeletal muscle (d), and cardiac muscle (e) from male mice were stained with H&E to visualize the structural differences among different groups (*n* = 6–10). Data are presented as mean ± *SD*. ^*^ *p* < .05, ^**^ *p* < .01^***^ *p* < .001. (f) Representative Western blots show the expression of myostatin (Mstn) in muscles, and the statistical significance of the results was demonstrated (*n* = 6). Data are presented as mean ± *SD*. ***p < .001. See also Figure S2 and Table S1

To further investigate the structural differences, some remarkably altered organs and tissues were stained with hematoxylin–eosin (H&E). The results revealed prominent signs of atrophy in KO + CD mice compared to WT + CD mice: lipid droplets in white adipose tissue (WAT) and brown adipose tissue (BAT) were much smaller (Figure 2a,b); the liver cells were smaller, judging by the higher density of the nucleus in the same sectional view (Figure 2c); the muscle fiber crossed‐sectional diameter was shorter in the skeletal muscle and cardiac muscle (Figure 2d,e); the volume of seminiferous tubule was decreased, and the hierarchical structure was not obvious in the testis (Figure S2d); the number of lymphoid nodule marginal zone cells was decreased in the spleen (Figure S2e); and the number of goblet cells and secretory glands decreased, accompanied with decreased excreta (Figure S2f); the intestinal villi are misaligned and shorter, and the luminal linings are thinner (Figure S2g). The high‐fat diet significantly reversed these atrophy features (Figure 2 and Figure S2). It was reported recently that SIRT6 can block myostatin expression and the development of muscle atrophy (Samant et al., 2017). The high‐fat diet efficiently attenuated the overexpression of myostatin caused by the SIRT6 deficiency (Figure 2f). The above results suggested that the high‐fat diet can reverse SIRT6 KO‐induced multi‐organ atrophy and tissue atrophy.

**Figure 3:**
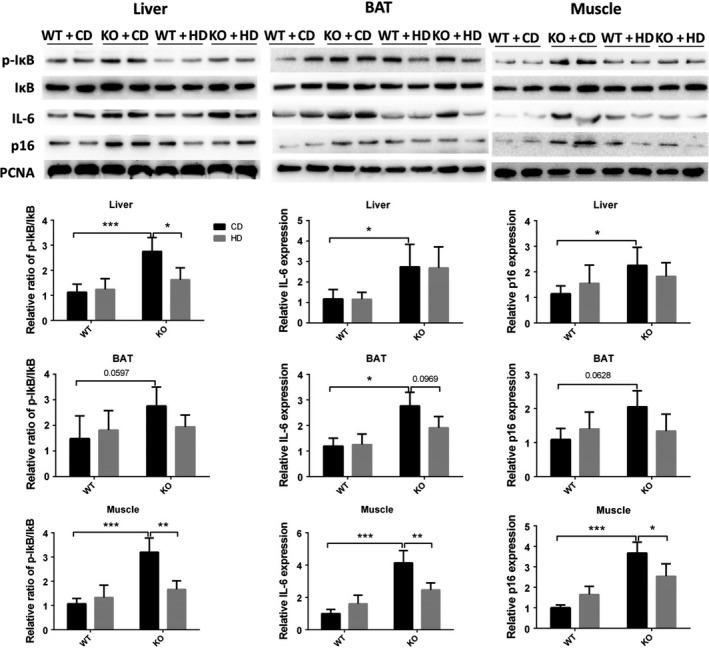
The high‐fat diet inhibits SIRT6 KO‐induced activation of NF‐κB in muscle. Representative Western blots illustrate the phosphorylation of IκB and expression of IκB, IL‐6, and p16 in the liver, BAT, and muscle. PCNA served as a reference (*n* = 6). Data are presented as mean ± *SD*. *p < .05, ***p < .001

It was reported that SIRT6 attenuates NF‐κB signaling via deacetylation of histone H3 Lys9 (H3K9) in chromatin, and hyperactive NF‐κB signaling may contribute to premature and normal aging (Kawahara et al., 2009). We did detect increased phosphorylation of IκB or overexpression of IL‐6 and p16 in the liver, BAT, and muscle of KO mice, and the high‐fat diet attenuated only the activation of NF‐κB signaling in muscle (Figure 3).

### 2.3 The metabolic pattern is changed, and a gene expression profile is partially normalized by the high‐fat diet in SIRT6 KO mice

**Figure 4:**
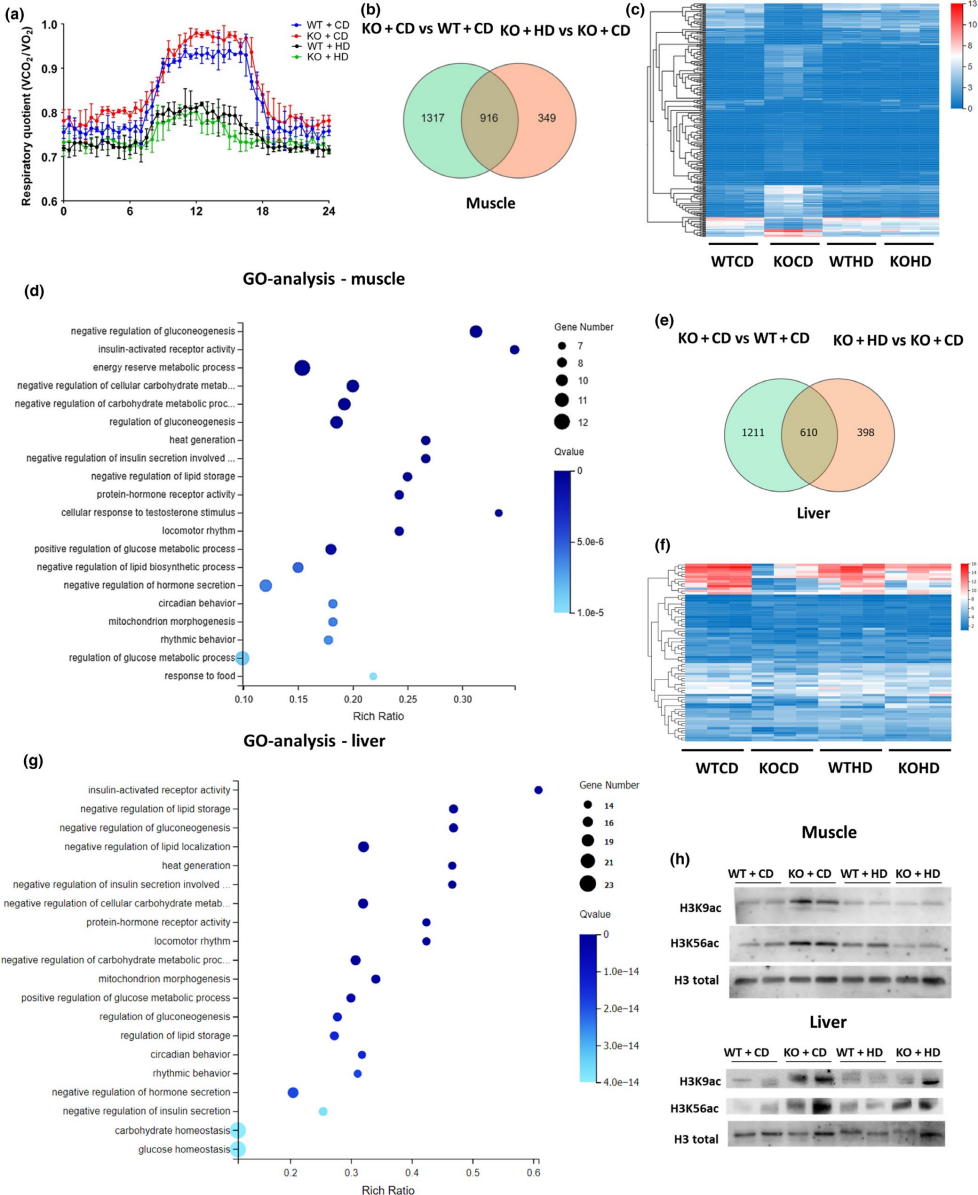
The metabolic pattern and gene expression profile are changed by the high‐fat diet in SIRT6 KO mice. (a) The respiratory quotient (VCO_2_/VO_2_) during 24 hr in each group was determined by means of metabolic chambers (*n* = 3). Whole‐genome sequencing was performed on the liver and muscle tissue samples from male mice. (b) The number of differentially expressed genes in the overlap between gene sets corresponding to genotypes and diets in muscle tissue. The screening criteria were *p* < .001 and the expression change greater than fourfold. (c) The heat map that was built from hierarchical clustering of 271 highly differentially expressed genes (*p* < .0001, fold change more than 8) out of the 916 genes in the overlap between the two sets of differentially expressed genes in muscle tissue. The heat map presents the gene expression patterns of different groups. (d) GO analysis of the 271 genes—out of the 916 genes in the overlap—indicates the signaling cascades and other pathways that were involved. (e) The number of differentially expressed genes in liver tissue. The screening criteria were *p* < .001 and the expression change greater than fourfold. (f) The heat map that was built from hierarchical clustering of 75 highly differentially expressed genes (*p* < .0001, fold change more than 8) out of the 610 genes in liver tissue. (g) GO analysis of the 75 genes indicates the signaling cascades and other pathways that were involved. (h) Representative blots show the acetylation level of histone 3 at lysine 9 and lysine 56 in muscle and liver tissues. See also Figure S3

To determine the direct action of the high‐fat diet on the metabolic pattern, we monitored the respiratory quotient (RQ). The high‐fat diet effectively induced a switch of the metabolic pattern from glucose metabolism to lipid metabolism both in WT and in KO mice because the average RQ decreased. Of note, the RQ in KO + CD mice was a little higher than that in WT + CD mice during the night (Figure 4a).

To further elucidate the molecular mechanism behind these changes, we performed RNA sequencing on the muscle tissue and liver tissue of male mice. The SIRT6 KO yielded a large number of differentially expressed genes, whereas the high‐fat diet decreased the differences between the WT and KO groups. As for the muscle tissue, 916 genes were found in the overlap between two gene sets: 2,233 differentially expressed genes between groups WT + CD and KO + CD and 1,265 differentially expressed genes between groups KO + HD and KO + CD (Figure 4b). To further narrow down the gene set, 271 genes were screened out of the 916 genes with a higher standard. The difference in the gene expression profile between WT and KO mice was much smaller after treatment with the high‐fat diet (Figure 4c). Gene Ontology (GO) analysis and Kyoto Encyclopedia of Genes and Genomes (KEGG) pathway analysis indicated that these genes were mainly involved in metabolic processes, including regulation of gluconeogenesis, insulin‐activated receptor activity as well as lipid and carbohydrate homeostasis (Figure 4d and Figure S3a). Moreover in liver tissues, 610 genes were found in the overlap between these two gene sets (Figure 4e). After increasing the screening standard, 75 highly differentially expressed genes were screened out of the 610 genes. High‐fat diet effectively decreased the expression difference caused by SIRT6 KO (Figure 4f). Gene ontology analysis and KEGG analysis also indicated that these genes were highly enriched in insulin signaling (Figure 4g and Figure S3b).iSIRT6 was reported to regulate target genes expression through working as a histone deacetylase. Many studies discovered the involvement of metabolism in epigenetic regulation. SIRT6 deficiency did increase the acetylation of histone 3 at lysine 9 an 56, high‐fat diet treatment significantly attenuate the levels in only in muscle tissue, which partially explains the different results of gene expression (Figure 4h and Figure S3c).

### 2.4 The high‐fat diet downregulates glycolysis by inhibiting insulin and IGF1–HIF1α signaling pathways in SIRT6 KO mice

**Figure 5:**
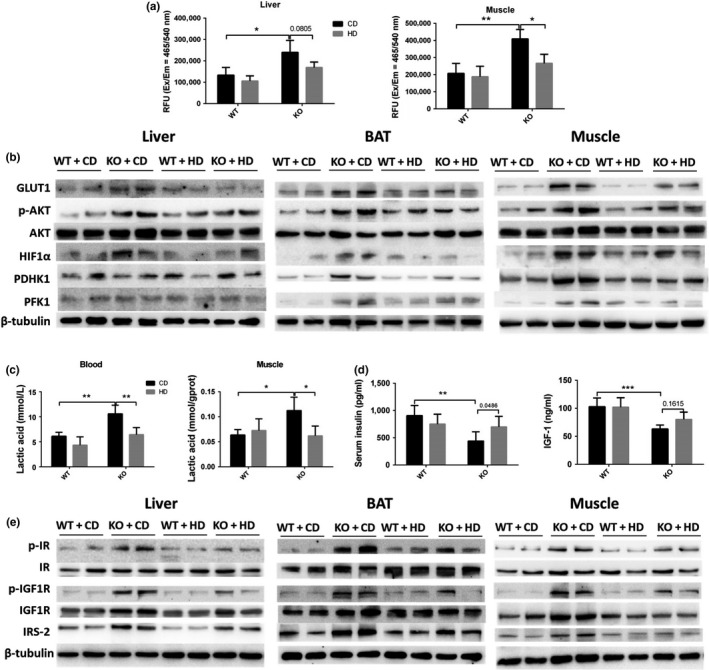
The high‐fat diet decreases glucose uptake, glycolysis, and activation of insulin and IGF1 signaling pathways in SIRT6 KO mice. (a) 2‐NBDG was injected intravenously (10 mg/kg body weight) after the starvation for 12 hr. The fluorescence signaling was tested in liver and muscle tissues, and the relative fluorescence units (RFU) were shown (*n* = 4). Data are presented as mean ± *SD*. *p < .05, **p < .01. (b) Representative Western blots show the amounts of GLUT1, AKT, p‐AKT, PDHK1, and PFK1 in the liver, BAT, and muscle from male mice. (c) The lactic acid levels in serum and muscle (*n* = 6). Data are presented as mean ± *SD*. *p < .05, **p < .01. (d) The serum levels of insulin and IGF1 were tested in male mice (*n* = 6 to 8). Data are presented as mean ± *SD*. **p < .01, ***p < .001. (e) Representative blots reveal the expression of insulin receptor (IR), phosphorylated IR (p‐IR), insulin‐like growth factor 1 receptor (IGF1R), phosphorylated IGF1R (p‐IGF1R), and insulin receptor substrate 2 (IRS2) in the liver, BAT, and muscle of male mice. See also Figure S4

It has been reported that SIRT6 can regulate glucose homeostasis, and SIRT6 deficiency results in hypoglycemia and increases glucose uptake by overactivation of AKT (Kim et al., 2010; Xiao et al., 2010; Zhong et al., 2010). In SIRT6 KO mice, the high‐fat diet decreased glucose uptake in muscle tissue and there was a mild but not significant decrease in liver tissue (Figure 5a). At the molecular level, the high‐fat diet attenuated the overexpression of GLUT1 in muscle and the upregulation of the phospho‐(p‐) AKT/total‐AKT ratio in all three types of tissue (Figure 5b and Figure S4a), suggesting that metabolic changes in muscle and BAT were the direct reason for the hypoglycemia and that the high‐fat diet may raise the blood glucose level by inhibiting glucose uptake in muscle and BAT.

It was reported that SIRT6 deficiency increases glycolysis and diminishes oxidation by activating hypoxia‐inducible transcription factor (HIF1α) signaling (Zhong et al., 2010). We obtained the same result, namely, SIRT6 KO increased the protein levels of HIF1α, pyruvate dehydrogenase kinase 1 (PDHK1), and phosphofructokinase 1 (PFK1) in muscle and BAT but not in the liver; however, the high‐fat diet effectively attenuated these changes only in muscle, and the HIF1α level was decreased by the high‐fat diet in BAT (Figure 5b and Figure S4a). Increased glycolysis and decreased oxidation should increase the lactic acid level. We did find increased lactic acid levels in the blood and muscle tissue of SIRT6 KO mice, and the high‐fat diet effectively decreased these lactic acid concentrations (Figure 5c).

As the key regulators of glucose metabolism and lipogenesis, insulin and IGF1 signaling cascades were affected by the SIRT6 KO and by the high‐fat diet. The latter increased the serum insulin concentration in the SIRT6 KO mice, but had no effect on the IGF1 level (Figure 5d). Furthermore, the increase in the phosphorylated insulin receptor (p‐IR) amount was attenuated in all three tissue types, and the p‐IGF1R/total‐IGF1R ratio was decreased in muscle and liver by the high‐fat diet. The expression of insulin receptor substrate 2 (IRS2) was attenuated only in muscle (Figure 5d and Figure S4b). These results suggested that the high‐fat diet can antagonize the hyperactivation of insulin and IGF1 signaling in a tissue‐dependent manner.

### 2.5 Fatty acids but not ketone bodies alleviate the SIRT6 KO‐induced excessive glycolysis and cell senescence

**Figure 6:**
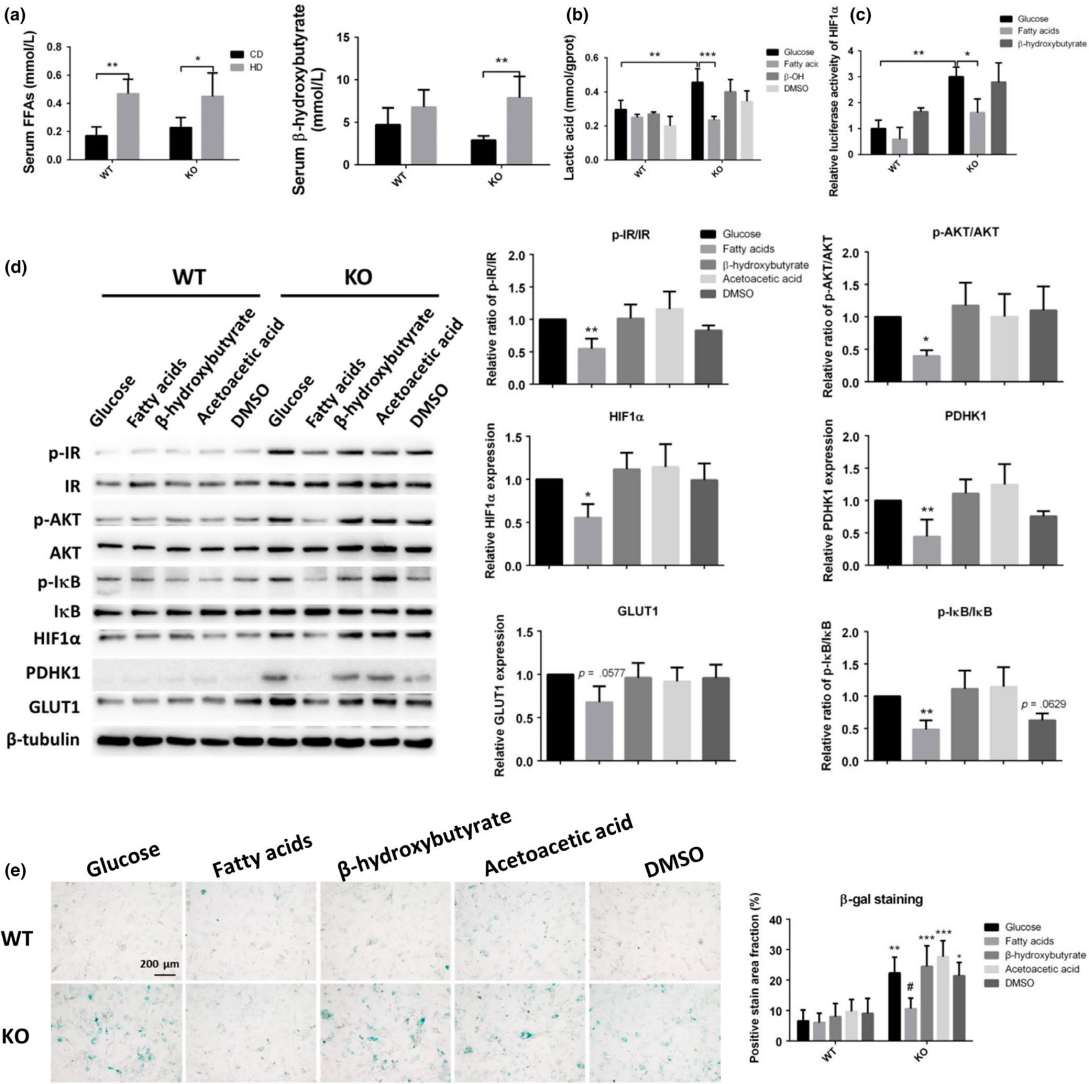
Fatty acids rather than ketone bodies inhibit glucose uptake and glycolysis in SIRT6 KO MEFs. (a) The plasma was collected from male mice at 3 hr postprandially, and the levels of FFAs and β‐hydroxybutyrate were measured next (*n* = 8). Data are presented as mean ± *SD*. *p < .05, **p < .01. (b) The lactic acid levels in WT and SIRT6 KO MEFs treated with fatty acids or ketone bodies were tested by an ELISA (*n* = 4). Data are presented as mean ± *SD*. *p < .05. (c) A luciferase reporter gene under the control of the HIF1α promoter was transfected into WT MEFs or SIRT6 KO MEFs, and these cells were cultured in a low‐glucose medium supplemented with glucose, fatty acids, or β‐hydroxybutyrate. Cell lysates were analyzed for luciferase activity (*n* = 3). Data are presented as mean ± *SD*. *p < .05, **p < .01. (d) Representative Western blots and statistical results indicate the phosphorylation of IR, AKT, and IκB and expression of HIF1α, PDHK1, and GLUT1. β‐Tubulin served as a reference. *p < .05, **p < .01. (e) WT MEFs or SIRT6 KO MEFs were cultured in the low‐glucose medium supplemented with glucose, fatty acids, or β‐hydroxybutyrate and were subjected to β‐Gal staining to demonstrate the cell senescence (*n* = 3). *p < .05, **p < .01, ***p < .001 compared with WT under same culture medium; ^#^ *p* < .05 compared with KO under high glucose medium. See also Figure S5

The high‐fat diet significantly increased the blood levels of free fatty acids (FFAs) and ketone bodies in KO mice (Figure 6a). Next, to test whether fatty acids or ketone bodies are the effector compounds in this process, we exposed mouse embryonic fibroblasts (MEFs) to a low‐glucose medium supplemented with glucose, mixed fatty acids, β‐hydroxybutyrate, or acetoacetic acid. Supplementation with fatty acids decreased the excessive expression of GLUT1 and PDHK1 and phosphorylation of IR and AKT. There was no significant change after treatment with two kinds of ketone bodies. The low‐glucose medium decreased the phosphorylation of IR and expression of GLUT1 but had no effect on PDHK1 and AKT activation (Figure 6a). Similarly, only fatty acids decreased the lactic acid level in SIRT6 KO MEFs (Figure 6b). As for the HIF1α signaling, fatty acids but not ketone bodies decreased the transcriptional activity of Hif1α in SIRT6 KO MEFs (Figure 6c). At the molecular level, fatty acids but not ketone bodies inhibited the phosphorylation of IR, AKT, and IκB and decreased the protein levels of HIF1α, PDHK1, and GLUT1 (Figure 6d). As for the cell senescence, fatty acids but not ketone bodies decreased the positive signal of β‐Gal staining in SIRT6 KO MEFs (Figure 6e).

To determine whether the influence of fatty acids on glycolysis is fatty‐acid type dependent, we used lauric acid, palmitic acid, oleic acid, and a mixture of fatty acids to treat MEFs separately. We did not find any differences among the different fatty‐acid treatments (Figure S5). These data suggested that the fatty acid supplement but not ketone bodies attenuated the excessive glycolysis caused by the SIRT6 deficiency.

### 2.6 Fatty acids inhibit excessive glycolysis partially through PI3K signaling

**Figure 7:**
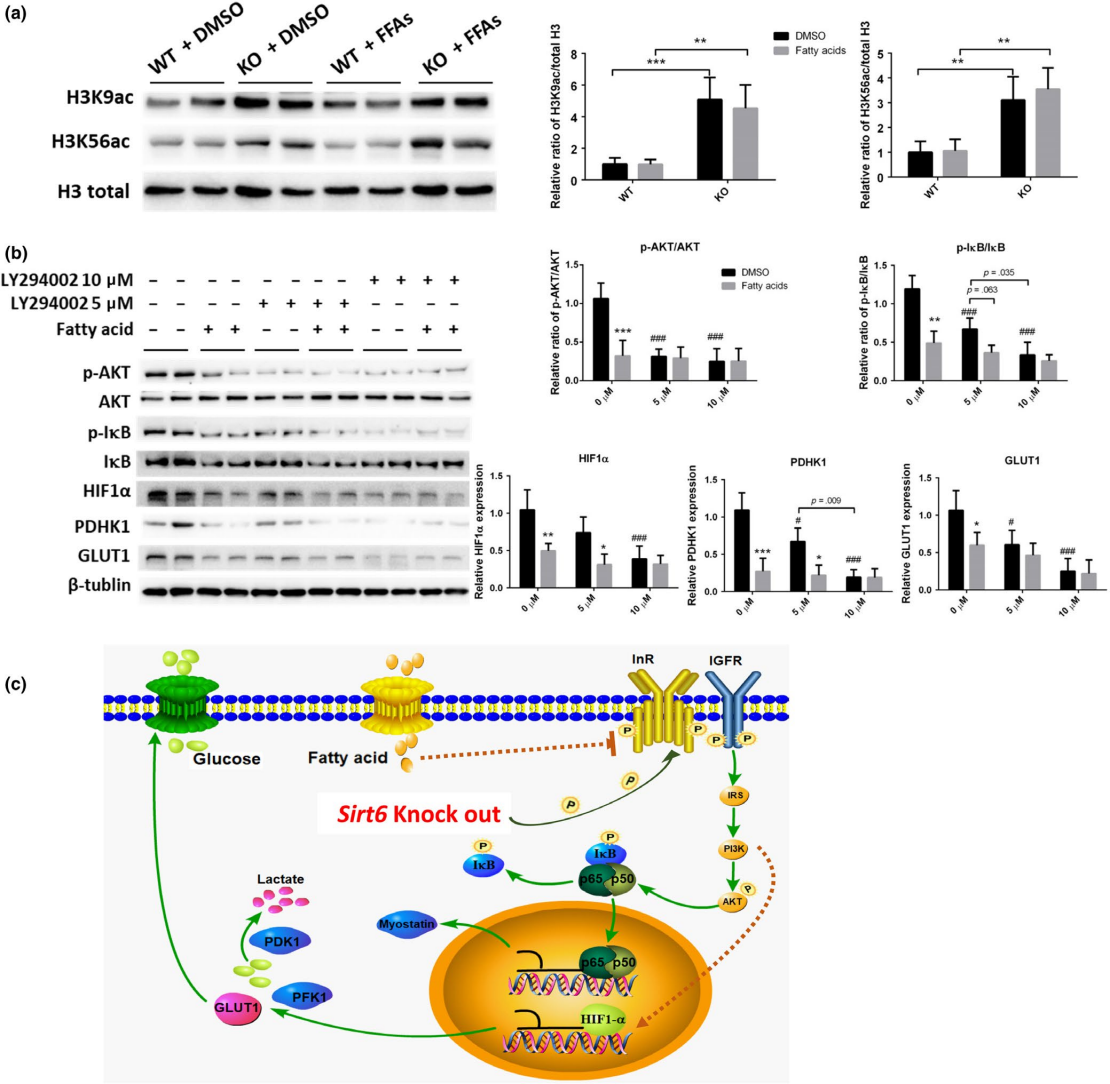
The PI3K inhibitor antagonizes the influence of fatty acids on SIRT6 KO MEFs. SIRT6 WT and KO MEFs were cultured with the medium supplemented with mixed fatty acids or DMSO. (a) Representative blots show the acetylation level of histone 3 at lysine 3 and lysine 56 and the statistical results are shown (*n* = 3). **p < .01, ***p < .001. (b) SIRT6 KO MEFs were treated with a PI3K inhibitor (LY294002) at 5 and 10 μM separately and with insulin at 17 μM. Representative blots and statistical results show the amounts of p‐AKT, AKT, p‐IκB, IκB, HIF1α, PDHK1, and GLUT1 (*n* = 4). *p < .05, **p < .01, ***p < .001 versus DMSO under same amount of inhibitor; ^###^ *p* < .001 versus no inhibitor group under DMSO treatment. (c) A proposed mechanism of the SIRT6 KO‐induced abnormalities and the reversing effect of the high‐fat diet: SIRT6 deficiency induces overactivation of IR and IGF1R, subsequently increasing the expression of IRS and phosphorylation of AKT, which increases phosphorylation of IκB. p‐IκB separates from the NF‐κB complex, thereby promoting the translocation of p65 and p50 into the nucleus to increase the expression of myostatin and other aging‐related genes. Upregulated HIF1α raises the expression of PDHK1, PFK1, and GLUT1, which strengthen glucose uptake and glycolysis. The high‐fat diet provides fatty acids and increases the circulating FFA levels in the blood and effectively inhibits insulin‐ and IGF1 signaling‐dependent glucose uptake and glycolysis. This mechanism may be the main reason for the reversal of the SIRT6 KO‐induced abnormalities by the high‐fat diet

As shown in Figure 4h, high‐fat diet made epigenetic changes in histone H3. In MEFs, high fatty acid culture did not change the acetylation levels of histone H3 at lysine 9 and 56, which were significantly increased by SIRT6 deficiency (Figure 7a). It has been reported that insulin signaling involves IRS1, IRS2, and the PI3K cascade activating AKT, which enhances glucose uptake (Chang, Chiang, & Saltiel, 2004; Cross, Alessi, Cohen, Andjelkovich, & Hemmings, 1995). To figure out whether the high‐fat diet acted through this pathway, a PI3K inhibitor (LY294002) was employed to test whether the inactivation of PI3K activity would block the action of the high‐fatty‐acid medium. Separately, 5 or 10 μM inhibitor was added into the high‐fatty‐acid medium to treat the cells for 48 hr. We found that the effect of fatty acids on AKT and IκB was attenuated by 5 μM inhibitor. During the 10 μM inhibitor treatment, the effect of fatty acids was totally blocked (Figure 7b). These results indicated that the influence of fatty acids on SIRT6 KO‐induced excessive glycolysis in MEFs was PI3K dependent (Figure 7c).

## 3 DISCUSSION

SIRT6 was reported to take part in multiple essential biological processes, including DNA repair, inflammation, apoptosis, and glucose and lipid metabolism (Kugel & Mostoslavsky, 2014). SIRT6 deficiency in mice causes a severe metabolic disorder, which leads to multi‐organ atrophy, premature aging features, and death within 1 month. Recently, it was reported that SIRT6 can block myostatin expression and SIRT6 deficiency‐induced muscle atrophy (Samant et al., 2017). In our experiments, the high‐fat diet effectively reversed the SIRT6 KO‐induced shortening of the lifespan, multi‐organ atrophy, premature aging, and metabolic abnormalities both in male and female mice.

According to the weight of various organs, SIRT6 KO‐induced organ atrophy is not specific, and the high‐fat diet effectively improved the state of almost all the atrophic organs and tissues (Table S1). Especially, the improvement of small intestine promotes nutrient assimilation, further increasing the blood glucose level. The molecular changes induced by high‐fat diet are different among liver, muscle, and BAT tissue (Figure 3 and Figure 5). Overall, the rescue effect of high‐fat diet is most prominent on muscle tissues, which highlight the major role of muscle in metabolism homeostasis. The SIRT6 KO induced severe hypoglycemia resulting from excessive glucose uptake and anaerobic glycolysis, which also promoted overutilization of stored lipids and glycogen. Compared to the normal rodent diet (high‐carbohydrate diet), the high‐fat diet provides more fatty acids as a source of energy and substrates of other essential biological phenomena at the same calorie intake. It was demonstrated that fatty acids and glucose compete for oxidation (Randle, Garland, Hales, & Newsholme, 1963). Given that in SIRT6 KO mice there was some defect in aerobic glucose metabolism, fatty acids might be a better energy source. Hypoglycemia was reported to be a direct cause of early death of SIRT6 KO mice (Mostoslavsky et al., 2006), and the high‐fat diet can increase the blood glucose level by inhibiting glucose uptake. However, the rescue effect is partial as the survival mice still died within 35 weeks. We speculate the early death results from hypoglycemia as the blood glucose levels of these survival mice are around half of the WT mice at the same age (data not shown). On the other hand, the supplementation with glucose in water did increase the blood glucose level but did not extend the shortened lifespan, suggesting that hypoglycemia is not the primary cause of early death and only energy source supplementation is not sufficient to rescue. Furthermore, glucose supplementation might produce adverse effect on metabolic homeostasis because of increased lactic acid level induced by excessive glycolysis. Glucose supplement did abrogate the rescue effect of reverse‐transcriptase inhibitors on SIRT6 KO‐induced shortened lifespan (Simon et al., 2019). Intriguingly, it has been reported that glucose supplement attenuated SIRT6 KO‐induced mutant phenotypes and increased shortened lifespan, which is different to what we got (Xiao et al., 2010). We speculate this discrepancy might result from the different mouse strains that are used to make SIRT6 mutant mice. A ketogenic diet (very little carbohydrate in the diet) also extended the shortened lifespan, and we did not note a better effect in comparison with the high‐fat diet (data not shown).

High‐fat diet (HFD) treatment affects the expression of a panoply of SIRT6‐targeted genes based on the RNA‐seq of liver and muscle tissues. High‐fat diet reduces acetyl‐CoA level and histone acetylation at multiple residues in a tissue‐specific manner (Carrer et al., 2017). As for the histone acetylation regulation, HFD significantly decreased the acetylation level only in muscle tissues, which partially explains the different molecular changes observed in muscle and liver tissues.

Insulin signaling is the key pathway regulating glucose and lipid metabolism (Prentki, Matschinsky, & Madiraju, 2013; Saltiel, 2016). The SIRT6 deficiency induced overactivation of IR and IGF1R in the heart tissue even without any change of blood insulin or glucose levels, which suggests that phosphorylation of IR and IGF1R is regulated by SIRT6 but not secondary effect of blood glucose or insulin (Sundaresan et al., 2012). It has been demonstrated that a high‐fat diet can decrease p‐IR levels and inhibit the activation of AKT, and also decrease glucose uptake and promote insulin resistance (Nguyen et al., 2005; Ramalho et al., 2017; Singh et al., 2016; Sodhi et al., 2012). Although insulin resistance is widely accepted as a risk factor of diabetes, it is a necessary response to evolutionary pressure when the main challenges to survival are not obesity and diabetes, but acute trauma and prolonged fasting (Soeters & Soeters, 2012). In our experiments, the high‐fat diet effectively attenuated the activation of IR and IGF1R, thereby decreasing glucose absorption and glycolysis and alleviating the atrophy of multiple organs and tissues. These findings also proved that the metabolic disorders are the primary cause of SIRT6 KO‐induced abnormalities. The molecular changes in BAT, muscle, and liver during the high‐fat diet were different, and the differences might have resulted from some tissue‐specific expression of genes inducing different responses to the SIRT6 KO and high‐fat diet. These differences also point to the different roles of these tissues under externally imposed metabolic stress.

High‐fat diet increased blood levels of fatty acids and β‐hydroxybutyrate in KO mice. Ketone bodies were reported to function as a metabolic and signaling mediator, not only as an alternative energy source (Puchalska & Crawford, 2017). Our results suggest that fatty acids, rather than ketone bodies, were the key factor responsible for the effect of the high‐fat diet because addition of fatty acids per se had a similar effect on SIRT6 KO MEFs. It is known that fatty acids can act as intracellular signaling molecules and participate in multiple processes (Papackova & Cahova, 2015). The notion that fatty acids can inhibit glucose uptake and insulin sensitivity dates back to the discovery that fatty acids can have this kind of effect in heart muscle (Randle et al., 1963). More and more evidence supports multiple hypotheses about how fatty acids decrease insulin sensitivity (Griffin et al., 1999; Nguyen et al., 2005; Szendroedi et al., 2014; Yu et al., 2002). In our study, fatty acids effectively inhibited the phosphorylation of IR and IGF1R and activation of the downstream factors, thereby decreasing glucose uptake, glycolysis, and production of lactic acid. Furthermore, fatty acids decreased protein level and inhibited the transcriptional activity of HIF1α, which is a key regulator of glycolysis. It was reported that SIRT6 KO increase the translation and stability of HIF1α and the mechanism remains unknown (Zhong et al., 2010). It has been reported that PI3K and AKT signaling stimulates mTOR activity, which stimulates translation of HIF1α mRNA into protein by phosphorylation of eIF4E binding protein (Laughner, Taghavi, Chiles, Mahon, & Semenza, 2001; H. Zhong et al., 2000). We speculate SIRT6 regulates HIF1α level at least partially by PI3K‐AKT signaling, as PI3K inhibitor could abrogate the changes induced by SIRT6 KO (Figure 7b). Furthermore, inhibition of PI3K abrogated the action of fatty acids on the SIRT6 KO phenotype, and the effect depended on the inhibitor concentration, proving that the influence of fatty acids on glucose uptake and glycolysis was partially dependent on insulin signaling. Nevertheless, how fatty acids regulate the activity of IR and IGF1R, as seen in this study, remains to be determined. Fatty acids do not change the acetylation levels of H3K9 and H3K56, which were increased a lot by SIRT6 deficiency. Several pathways have been reported before explaining how fatty acids inhibit insulin sensitivity, for example, through diacylglycerol and ceramides (Samuel, Petersen, & Shulman, 2010). The effect of fatty acids was independent of the fatty‐acid type in our study and was seen only when SIRT6 was knocked out. One possible explanation is that fatty acids can inhibit the expression or activity of glycolysis‐related enzymes only when the glycolytic level reaches a threshold. The other possibility is that the SIRT6 KO induces changes in the expression of some genes or in signaling cascades that may strengthen the influence of fatty acids. More research needs to be done to elucidate this.

Increased glycolytic capacity should be beneficial under some conditions, such as nutritional stress or rapid cellular proliferation, but sustained overactivation of glycolysis is detrimental, judging by the aberrations in SIRT6 KO mice. Therefore, the switch from the normal state to increased glycolysis should be strictly controlled. In this study, we proposed a high‐fat diet as an effective strategy to intervene in the switching of this process.

This study is the first (a) to prove that the SIRT6 KO‐induced phenotype can be improved through a dietary intervention and (b) to thoroughly elucidate the role of SIRT6 in the regulation of glucose and lipid metabolism. In addition, our study provides evidence that a glucose metabolism disorder can be efficiently treated by targeting lipid metabolism and fatty acid can function as a signaling regulator, thus offering a new basis for novel therapeutic strategies against metabolic disorders and atrophy‐related diseases.

## 4 EXPERIMENTAL PROCEDURES

### 4.1 Animal husbandry and dietary interventions

SIRT6*^tm1.1Cxd^* mice with a 129Sv background were acquired from the Jackson Laboratory (Bar Harbor, ME, USA). The mice were maintained under semi‐specific pathogen‐free (SPF) conditions. The following primers were used for genotyping: forward, 5′‐AGTGAGGGGCTAATGGGAAC‐3′; reverse, 5′‐AACCCACCTCTCTCCCCTAA‐3′. The SIRT6 KO‐associated PCR product is 453 bp long, whereas the WT PCR product's size is 399 bp. Three‐week‐old SIRT6 KO and WT mice were fed a control standard AIN‐93G diet (abbreviated as CD; 64% carbohydrates, 19% protein, and 17% fat) or a high‐fat diet consisting of AIN‐93G with 65% of calories from fat, principally hydrogenated coconut oil (16% carbohydrates, 19% protein, and 65% fat; abbreviated as HD). Because of high‐fat proportion, high‐fat food is damper than the control food.

In consideration of weakness and smaller body size of KO mice, the food was supplied on ground and the glucose water or normal water was provided in hanging bottle, which is accessible to KO mice. To ensure that enough food was supplied, food intake was measured to determine the daily food consumption required by WT and KO mice on different days separately. The amounts of the high‐fat diet and standard control diet were calculated as caloric intake per day per body weight in 4‐week‐old KO mice or WT mice. All the measurements were done at 4 weeks of age. Organ weights were measured after blood collection. All the animals were singly housed from 3 weeks of age with a plaything to allow for acclimation to the animal facility. For a lifespan experiment, SIRT6 KO mice were fed with the continuous standard control diet or high‐fat diet until death. Unless stated otherwise, blood samples were collected after 3 hr of fasting at approximately 6 hr into the light cycle. Body weight was monitored twice a week. Animal rooms were maintained at 23°C on a 12 hr light/dark cycle. All animal protocols were approved by the Institutional Animal Care and Use Committee (IACUC) of Tsinghua University.

### 4.2 Cell culture

Please refer to Appendix S1 to get more details.

### 4.3 Plasma analysis

Please refer to Appendix S1 to get more details.

### 4.4 Body composition and bone density

Please refer to Appendix S1 to get more details.

### 4.5 H&E staining

Please refer to Appendix S1 to get more details.

### 4.6 A luciferase reporter assay

Please refer to Appendix S1 to get more details.

### 4.7 Microarray analysis

Please refer to Appendix S1 to get more details.

### 4.8 Metabolic assessment

Please refer to Appendix S1 to get more details.

### 4.9 Glucose uptake assay

Please refer to Appendix S1 to get more details.

### 4.10 Western blotting

Please refer to Appendix S1 to get more details.

### 4.11 Statistical analysis

All the results are expressed as means ± *SD*. Comparisons among several groups were performed by two‐way ANOVA. Data were analyzed in Graph Pad Prism 6.0 software.

## CONFLICT OF INTEREST

Authors declare no competing interests.

## AUTHORS' CONTRIBUTION

Z.C.L. and Z.W. conceived the study; Z.C.L., K.X., and Z.W. designed the experiments; Z.C.L. and K.X. conducted most of the experiments and data analyses; Y.N.G., Y.Q.G., and L.P. performed animal feeding, dissection, and tissue staining. Y.Q. and Y.N.G. conducted staining analyses and quantification; Q.F.L., J.Q.N., and Z.W. contributed to the discussion and data interpretation; Z.C.L. and K.X. wrote the manuscript.

## Data Availability

All data are available in the manuscript or the supplementary materials. Correspondence and requests for materials should be addressed to corresponding author Z.W.
