# Supplementary material for: A high‐fat diet reverses metabolic disorders and premature aging by modulating insulin and IGF1 signaling in SIRT6 knockout mice
Source: Aging Cell. 2020 Jan 22;19(3):e13104. doi: 10.1111/acel.13104 (PMC7059164; doi:10.1111/acel.13104)
Supplement: Supplementary file 1 [file ACEL-19-e13104-s001.docx]

Appendix S1

Supplementary methods

**Microarray analysis**

Total RNA was extracted from liver and muscle samples using the Eastep Super RNA Extraction Kit (Promega, Madison, USA) according to the manufacturer’s instructions. RNA purity was checked on a NanoPhotometer spectrophotometer (IMPLEN, CA, USA), and RNA integrity was verified using the RNA Nano 6000 Assay Kit of the Bioanalyzer 2100 system (Agilent Technologies, CA, USA). Total-RNA samples (3 μg) were processed by Novogene Bioinformatics Institute for mRNA enrichment using poly-T oligo–conjugated magnetic beads. Clustering of the index-coded samples was performed on a cBot Cluster Generation System with the TruSeq PE Cluster Kit v3-cBot-HS (Illumina) according to the manufacturer’s instructions, and the libraries were sequenced on the Illumina HiSeq 4000 platform. HTSeq v0.6.1 was used to determine the read numbers mapped to each gene. Fragments per kilobase of exon per million fragments mapped (FPKM) of each gene were calculated based on the length of the gene and the count of reads mapped to this gene. Differential analysis of count data was carried out in the DESeq2 software. Genes with an adjusted P value less than 0.0001 and an expression fold change greater than 8 were selected as differentially expressed genes for subsequent GO and KEGG enrichment analyses.

**Metabolic assessment**

Metabolic rate of the mice was assessed in metabolic chambers (PhenoMaster, TSE systems, Germany) at the Laboratory Animal Research Center of Tsinghua University. Four-week-old mice were housed individually in metabolic chambers maintained at 23°C on a 12:12 hr light:dark cycle (light period 07:00–19:00 hr) with free access to water. Food supply was calculated according to body weight. All the mice were acclimated to the monitoring cages for 8 hr before recording. The concentrations of O2 and CO2 were determined by analyzing the air entering and leaving the chamber to calculate respiration. The sensors were calibrated against a standard gas mix containing defined quantities of O2, CO2, and N2. Constant airflow (0.6 L/min) was maintained in the chamber and was monitored by a mass-sensitive flow meter. Each chamber was analyzed for 3 min at 27 min intervals, and data were recorded for 72 hr total. The respiratory exchange ratio is the ratio of CO2 production to O2 consumption.

**Glucose uptake assay**

2-NBDG (APE×BIO, Catalog No. B6035) was injected intravenously through the tail vein into the mice (25-28 days old, 10 mg/kg bodyweight), which were starved for 12 hours before the injection. After 5 minutes, liver and muscle tissue samples (100mg) were dissected and lysed with light blocked on the ice immediately. The homogenate was then centrifuged at 4 ℃, 10000 rpm for 5 minutes. Supernatant was gained for plate reader (GLOMAX Multi Detection System, Promega, Madison, WI) detection at a wave length of Ex465nm/Em540nm.

**Western blotting**

Samples were prepared in middle RIPA lysis buffer (Biomiga, CA, USA), supplemented with a cocktail of protease (AbMole Bioscience, Houston, USA) and phosphatase inhibitors (Solarbio, Beijing, China). Samples containing equal amount of protein were run on 10% SDA-PAGE and then transferred to a polyvinylidene difluoride (PVDF) membrane (Millipore, Bedford, MA, USA). The membrane was blocked for 30 minutes in 5% skin milk (OXOID, Cheshire, UK) containing 0.1% fetal bovine serum (Gibco, Waltham, MA, USA). Blocked membranes were incubated overnight at 4°C with primary antibody diluent. The blots were then incubated with the appropriate horseradish peroxidase (HRP)-conjugated secondary antibody (Abcam, Cambridge, UK, USA) diluent at room temperature for 2 hr. Antibody bound protein was detected by SuperSignal West Pico Chemiluminescent Substrate (Thermo Scientific, Waltham, MA, USA). Protein signal was visualized using a Chemi Capture (CLINX, Shanghai, China) and signaling intensity was analysis by ImageJ software and normalized. Primary antibodies against the proteins were as follow: SIRT6 (#ab62739, Abcam), PCNA (#2586, Cell Signaling Technology, Danvers, MA, USA), Mstn (#AP2068a-400, Abgent, San Diege, CA, USA), p-IκB (#AP50216, Abgent), IκB (#AP2506a, Abgent), IL-6 (#12912, Cell Signaling Technology), p16 (ab220800, Abcam), GLUT1 (#12939, Cell Signaling Technology), p-AKT (#4060T, Cell Signaling Technology), AKT (#2920, Cell Signaling Technology), HIF1α (#14179, Cell Signaling Technology), PDHK1 (#3820, Cell Signaling Technology), PFK1 (#sc-377346, Santa Cruz Biotechnology, Dallas, Texas, USA), p-IR (#ab203278, Abcam), IR (#3025, Cell Signaling Technology), p-IGF1R (#3918, Cell Signaling Technology), IGF1R (#3018, Cell Signaling Technology), IRS-2 (#4502, Cell Signaling Technology), β-tubulin (#2128, Cell Signaling Technology)，Histone H3 (#4499, Cell Signaling Technology), Acetyl-Histone H3 (Lys56) (#4243, Cell Signaling Technology), Acetyl-Histone H3 (Lys9) (#9649, Cell Signaling Technology). Second antibodies: Goat anti-rabbit IgG and goat anti-mouse IgG (Santa Cruz Biotechnology).

**H&E staining**

All tissues were fixed in 4% paraformaldehyde (Servicebio, Wuhan, China) for 24 hr at 20-25°C and then embedded in paraffin. The samples were dehydrated, and 4 μm sections were studied. H&E staining was performed according to the manufacturer’s instructions (Sigma-Aldrich, Munich, Germany). Photographs were captured using a light microscope (Leica, Wetzlar, Germany). Zen 2.3 (blue edition, Carl Zeiss Microscopy GmbH, 2011) was used to quantify the size of lipid droplet and diameter of muscle fiber.

**A luciferase reporter assay**

MEFs were cotransfected with a HIF1α or c-JUN promoter-reporter plasmid and the pGMLR-CMV reporter plasmid (Yeasen, Shanghai, China) using Lipofectamine 2000 (Invitrogen) in OPTI-MEM (Gibco). After 24 hr incubation, firefly luciferase and Renilla luciferase activities of cells were evaluated with the Dual Luciferase Reporter Gene Assay Kit (Yeasen, Shanghai, China) on a GLOMAX Multi Detection System (Promega, Madison, WI, USA).

**Plasma analysis**

Blood collection was performed via cardiac puncture. Plasma was obtained after centrifugation at 1500 × g for 15 min at 4°C and was frozen at −80°C until thawing for an assay. The following plasma assays were conducted using kits: IGF-1 (CWBIO), β-HB (Cloud Clone Corp., Wuhan, China), and insulin (Cloud Clone Corp.). Glucose was quantified in whole blood with a glucose meter (Roche, West Sussex, UK). FFAs were quantitated on an automatic biochemical analyzer (HITACHI 7080, Tokyo, Japan). Plasma concentrations of triglycerides, CHO, high-density lipoprotein cholesterol, and low-density lipoprotein cholesterol were measured by means of an automatic biochemical immunity analyzer (COBAS 6000, Roche).

**Body composition and bone density**

Measurements of total fat body mass in live mice were performed by nuclear magnetic resonance (NMR) spectroscopy using a MesoMR23-060H-I imaging instrument (Shanghai Niumag Corporation, China). Bone density was determined by dual photon absorptiometry (Prodigy, General Electric, USA).

**Cell culture**

SIRT6 KO MEFs were isolated from KO embryos on embryonic day 13.5 and were genotyped by PCR. In brief, the embryonic head, limbs, and organs were removed, and the remaining tissues were cut into pieces smaller than 1 mm3. Tissue fragments were spread in a cell culture dish directly and were digested with 0.25% trypsin (Corning, Manassas, VA, USA) after 48 hr of cultivation. We filtered the cells with 70 μm strainers, and the MEFs were in the filtrate. MEFs were cultured in high-glucose DMEM (Gibco) supplemented with 10% of fetal bovine serum (FBS; Gibco) and 1% of a penicillin and streptomycin solution (Corning). The cells were treated with 200 μM fatty-acid mixture composed of lauric acid and palmitic acid (Sigma) as described previously (Nguyen et al., 2005). The final concentration of insulin (Sigma) was 17 μM. The cells were incubated with 5 or 10 μΜ PI3K inhibitor (LY294002) (Selleck, Shanghai, China) in the culture medium for 48 hr. The medium was refreshed every 16 hr. The cells were maintained at 5% CO2 and 37°C. Cells were lysed by RIPA lysis buffer (Thermo) with protease inhibitor (Thermo) and the lysates were collected, and total protein was quantified by means of the BCA Protein Quantification Kit (Thermo). Lactic acid levels were measured with the Lactic Acid Test Kit (BioAssay System, Hayward, CA, USA) according to the manufacturer’s instructions. Optical density at 530 nm was measured on a spectrophotometer (Alpha1506, LASPEC, Shanghai, China). SA-b-Gal staining was performed with the Senescence Cells Histochemical Staining Kit (Sigma). The Positively stained area was analyzed by ImageJ software（1.48v, Wayne Rasband, National Institutes of Health, USA）.

Supplementary figures


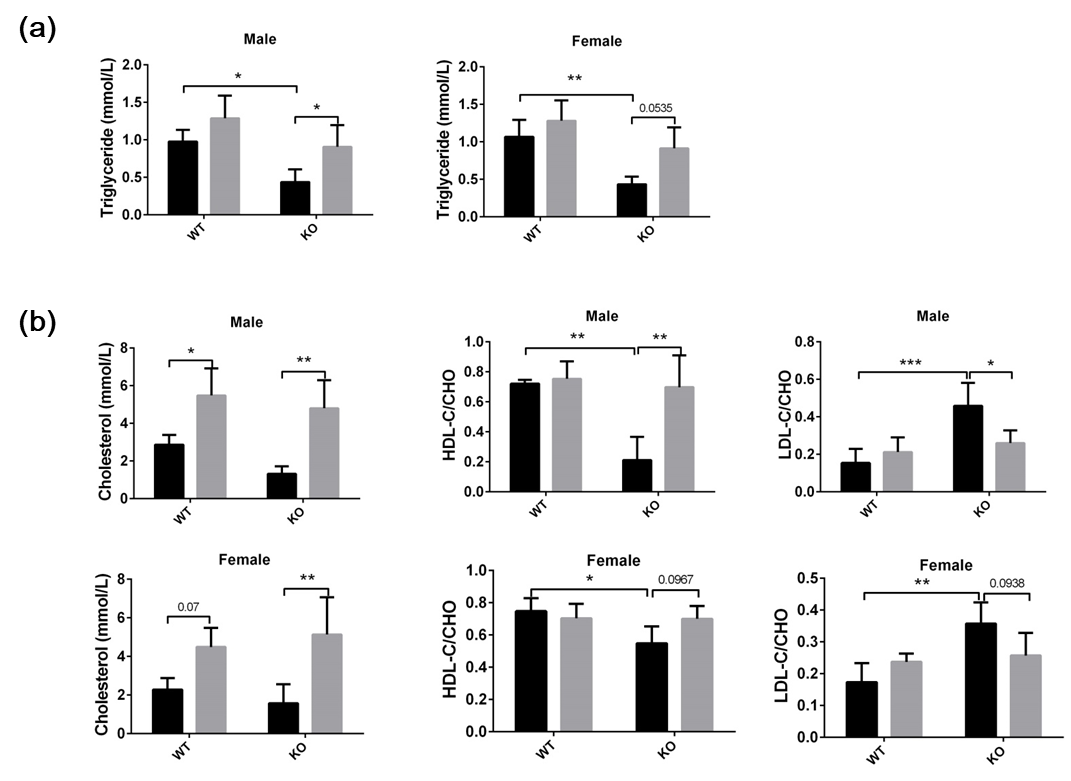


Fig. S1. The high fat diet increased blood lipid level in SIRT6 knock out mice. Related to Figure 1.

The serum levels of triglyceride (**a**), total cholesterol (CHO), high-density lipoprotein cholesterol (HDL-C)/CHO and low-density lipoprotein cholesterol (LDL-C)/CHO (**b**) were tested on both male and female mice (n=6-8). Data are represented as mean ± SD. ^*^ p<0.05, ^**^ p<0.01, ^***^ p<0.001.


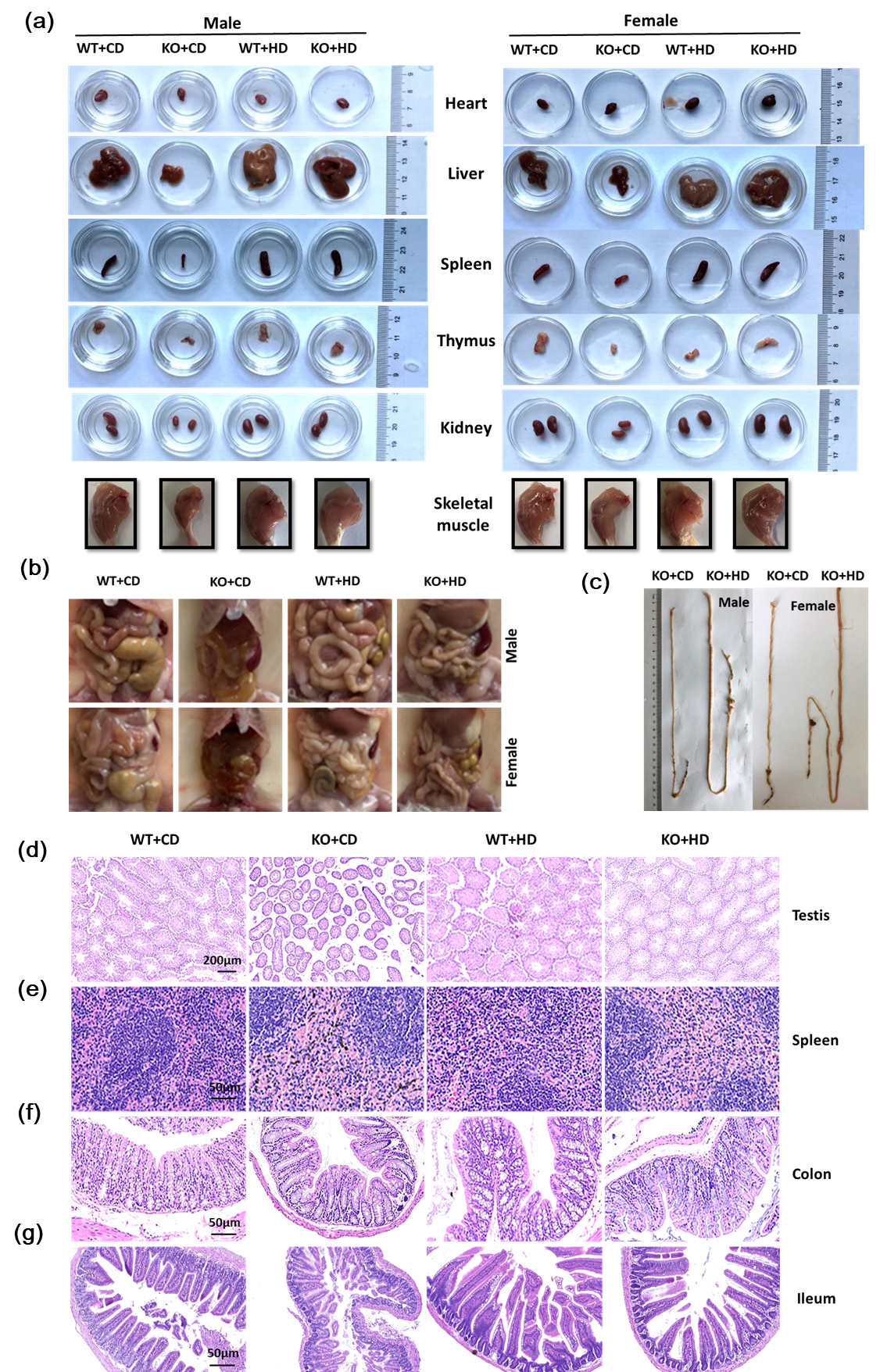


**Fig. S2. The high fat diet rescues the SIRT6 deficiency-induced multiple organ and tissues atrophy phenotypes. Related to Figure2.**

(**a**) Different group of male and female mice were dissected at around one month after one week high fat diet or normal control diet intervention. Different tissues and organs were weighted and compared. (**b**) Representative images of enterocoelia appearance were shown to compare the intestine and fat mass among different groups. (**c**) The intestine was taken out to compare the length and thickness between KO+CD mice and KO+HD mice. The testis tissues (**d**), spleen tissue (**e**), colon tissue (**f**), and ileum tissue (**g**) from male mice were stained with hematoxylin-eosin (H&E) satins to visualize the structure differences among different groups.


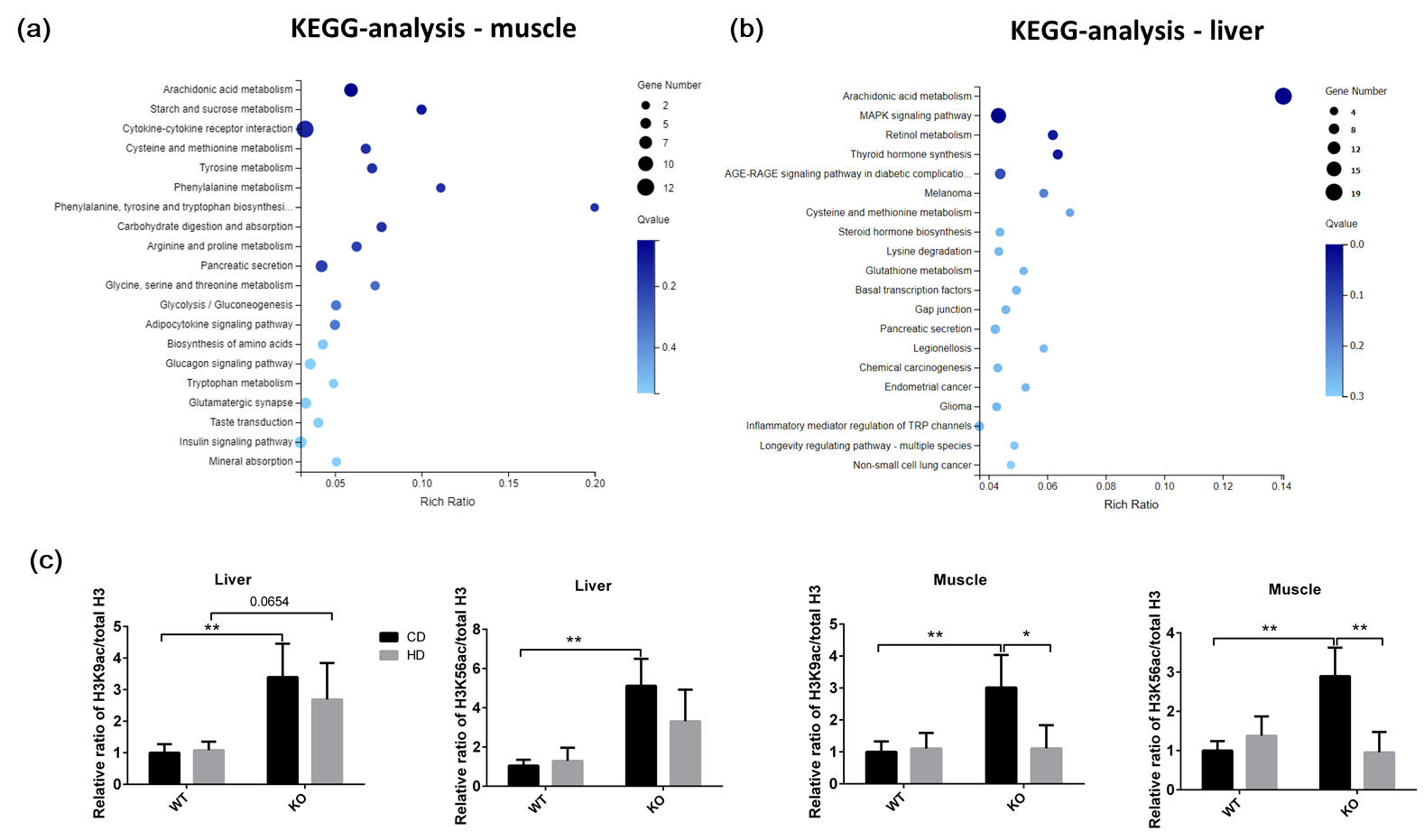


**Fig. S3. The gene expression profile was changed by high fat diet in SIRT6 deficient mice. Related to Figure 4.**

The differentially expressed genes in the overlap between gene sets corresponding to genotypes and diets were screened out separately in muscle and liver tissues. The screening criteria were p < 0.001 and the expression change greater than fourfold. (**a**) KEGG pathway analysis of the 271 genes out of the 916 genes in the overlap reveals biological processes that were involved in muscle tissue and (**b**) 75 genes out of the 610 genes in liver tissue. (**c**) The liver and muscle tissues were lysed to test the acetylation level of histone 3 at lysine 9 and lysine 56. The statistic results of the ratio of H3K9ac / total h3 and H3K56ac / total H3 were shown (n=4). Data are represented as mean ± SD. p<0.05, ^**^ p<0.01.


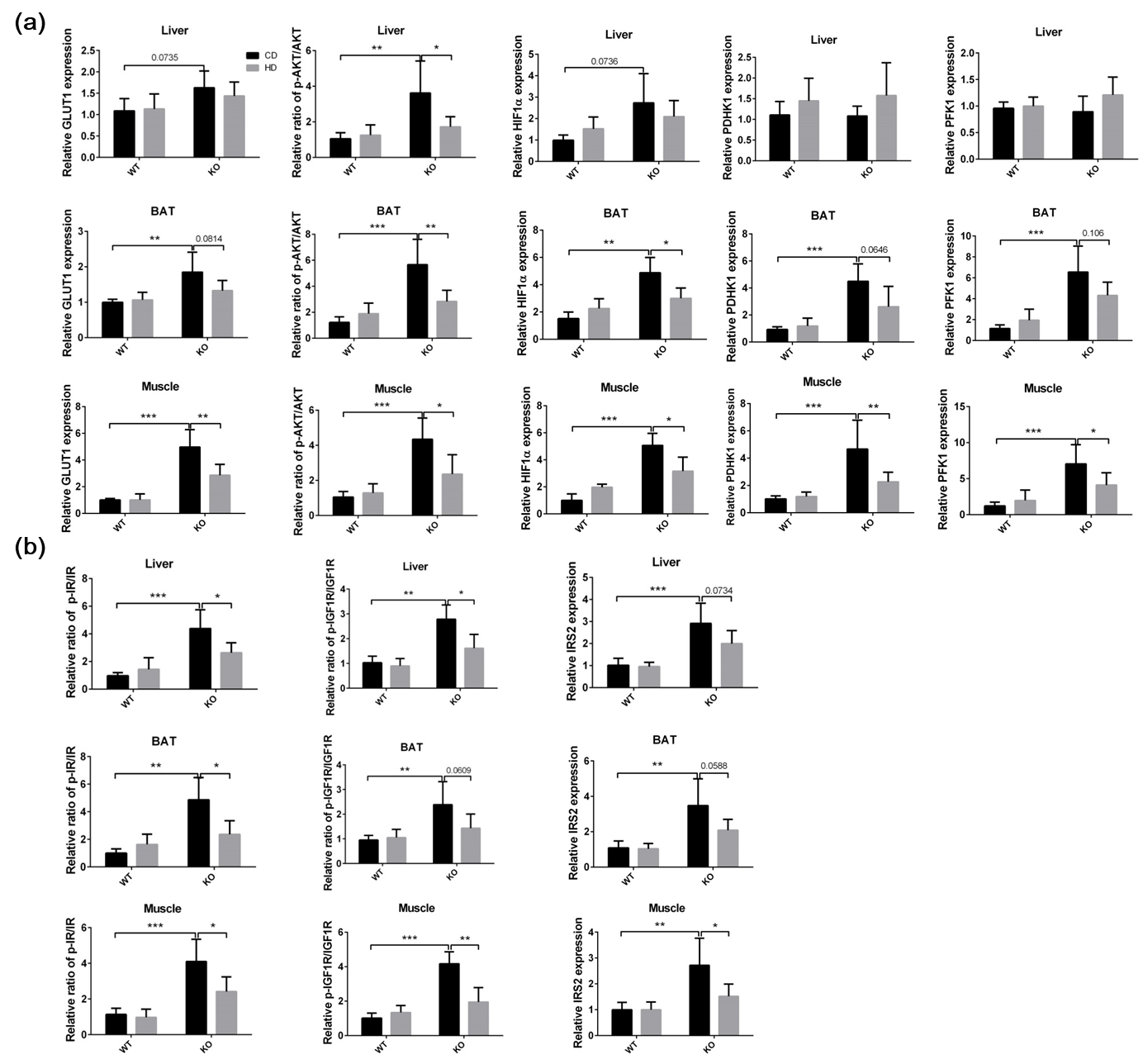


**Fig. S4. The high fat diet decreased expressions of glycolytic genes and activity of IR and IGF1R in SIRT6 deficiency mice. Related to Figure 5.**

The statistic results of the expression of glucose uptake and glycolysis related genes (**a**) and insulin/IGF1 signaling activity (**b**) (n=6). Data are represented as mean ± SD. ^*^ p<0.05, ^**^ p<0.01, ^***^ p<0.001.


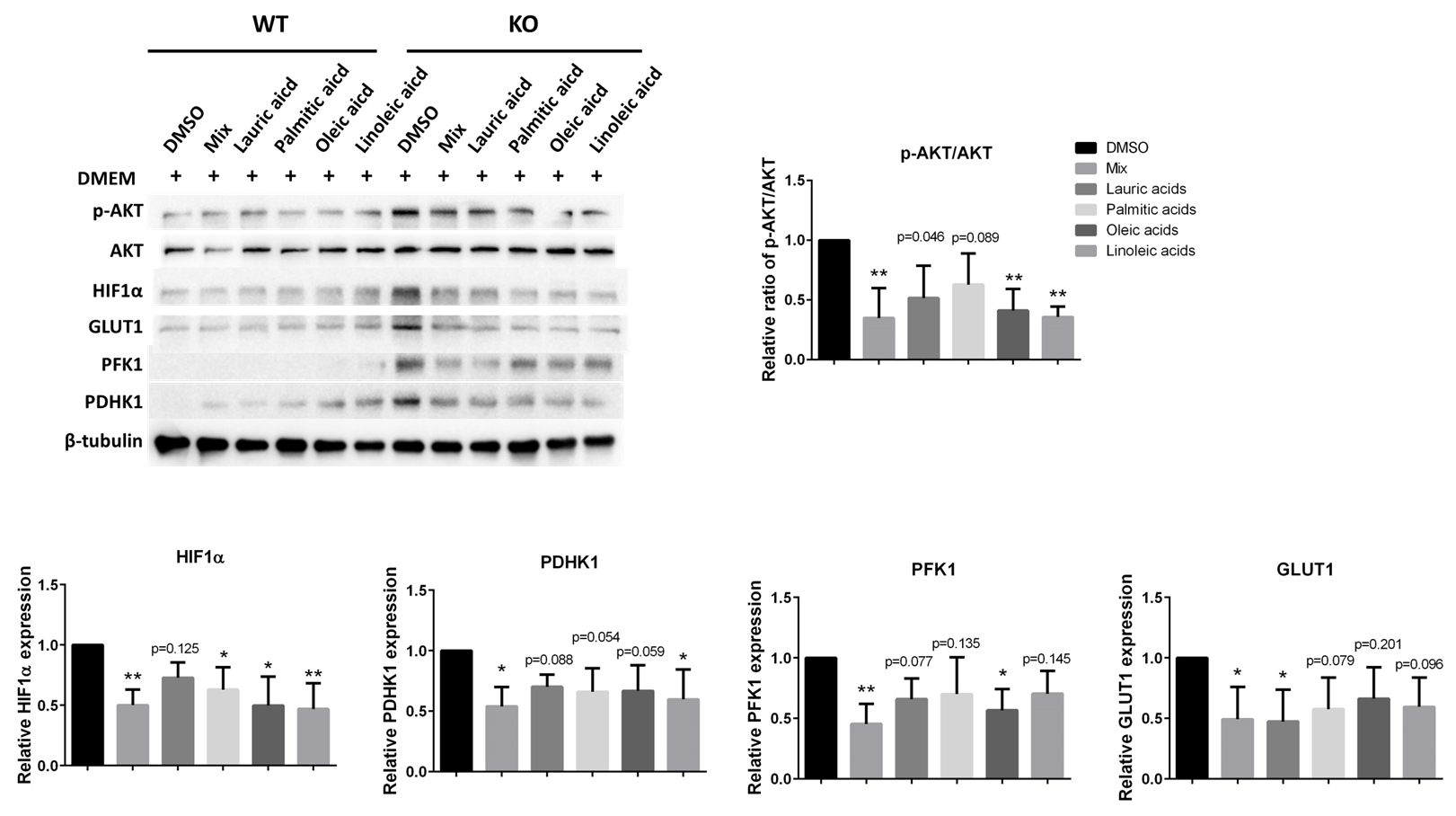


**Fig. S5. The fatty acid rather than ketone bodies could inhibit activation of IR and glycolysis. Related to Figure 6.**

The wild type and SIRT6 deficient MEF were treated with different kind of fatty acid for 24 hours. The protein levels of p-AKT, AKT, HIF1α, GLUT1, PDHK1 and PFK1 were tested by western blotting. β-tubulin was taken as reference. The statistic results of these proteins were shown (n=3). Data are represented as mean ± SD. ^*^ p<0.05, ^**^ p<0.01.


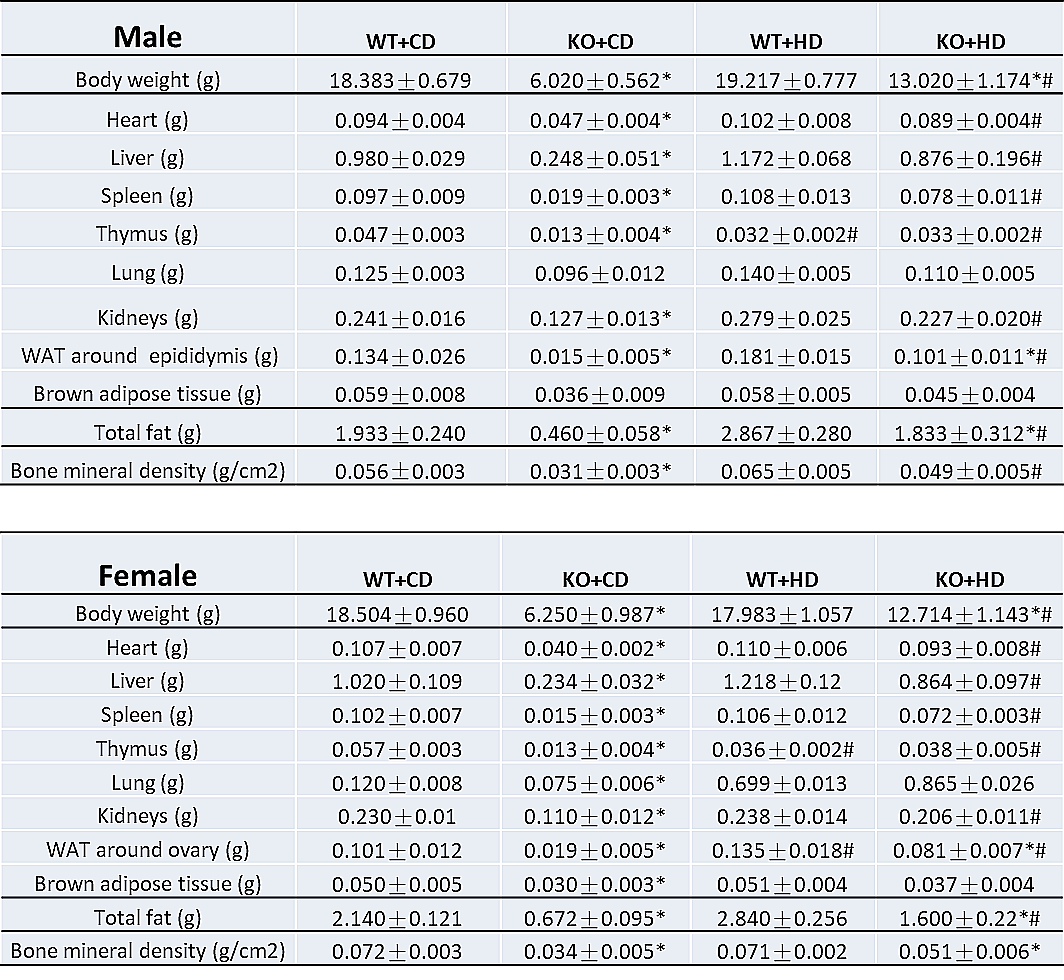


**Table S1. The weight of the whole body and different organs and tissues.**

Different group of mice were dissected at one month, and tissues and organs were obtained and weighted. Bone density scanner was used to get the total fat weight and bone mineral density (n=10-12). WAT: white adipose tissue.

* indicates a difference (*P*<0.05) between genotypes for the same diet.

# indicates a difference (*P*<0.05) diets for the same genotype.
